# Are miRNA-103, miRNA-107 and miRNA-122 Involved in the Prevention of Liver Steatosis Induced by Resveratrol?

**DOI:** 10.3390/nu9040360

**Published:** 2017-04-04

**Authors:** Ana Gracia, Alfredo Fernández-Quintela, Jonatan Miranda, Itziar Eseberri, Marcela González, María P. Portillo

**Affiliations:** 1 Nutrition and Obesity Group, Department of Nutrition and Food Science, University of the Basque Country (UPV/EHU) and Lucio Lascaray Research Institute, Vitoria 01006, Spain; anajadraque@gmail.com (A.G.); jonatan.miranda@ehu.eus (J.M.); itziareseberri@hotmail.com (I.E.); mariapuy.portillo@ehu.eus (M.P.P.); 2 CIBERobn Physiopathology of Obesity and Nutrition, Institute of Health Carlos III, Madrid 28029, Spain; 3 Nutrition and Food Science, Faculty of Biochemistry and Biological Sciences, National University of Litoral, Santa Fe 3000, Argentina; maidagon@fbcb.unl.edu.ar

**Keywords:** miRNA-103, miRNA-107, miRNA-122, steatosis, liver, resveratrol, rat

## Abstract

The aim of the present study was to determine whether the reduction in liver fat previously observed in our laboratory in a cohort of rats which had been fed an obesogenic diet was mediated by changes in the expression of microRNA (miRNA)-103-3p, miRNA-107-3p and miRNA-122-5p, which represent 70% of total miRNAs in the liver, as well as in their target genes. The expression of the three analysed miRNAs was reduced in rats treated with resveratrol. A reduction in sterol-regulatory element binding protein 1 (SREBP1) and an increase in carnitine palmitoyltransferase 1a (CPT1a) were observed in resveratrol-treated rats. No changes were found in fatty acid synthase (FAS). In cultured hepatocytes, SREBP1 protein was increased after the transfection of each miRNA. FAS protein expression was decreased after the transfection of miRNA-122-5p, and CPT1a protein was down-regulated by the over-expression of miRNA-107-3p. This study provides new evidences which show that *srebf1* is a target gene for miRNA-103-3p and miRNA-107-3p, *fasn* a target gene for miRNA-122-5p and *cpt1a* a target gene for miRNA-107-3p. Moreover, the reduction in liver steatosis induced by resveratrol in rats fed an obesegenic diet is mediated, at least in part, by the increase in CPT1a protein expression and activity, via a decrease in miRNA-107-3p expression.

## 1. Introduction

Excessive fat accumulation in liver is known as hepatic steatosis, which is the most benign form of non-alcoholic fatty liver disease (NAFLD). It is a major cause of chronic liver disease in Western societies. It encompasses a disease spectrum ranging from simple triglyceride accumulation in hepatocytes (hepatic steatosis; NAFL) to hepatic steatosis with inflammation (non-alcoholic steatohepatitis, NASH) [1]. This disorder is closely associated with obesity and insulin resistance [2]. Although the current treatment of liver steatosis is based on dietary energy restriction and physical activity [3,4], a great deal of attention has been paid in recent years to bioactive molecules, such as phenolic compounds present in foods and plants, which can represent complementary tools.

One of the most widely studied molecules is resveratrol (*trans*-3,5,4′-trihydroxystilbene), a phytoalexin occurring naturally in grapes, berries and peanuts [5,6]. Several studies on rats and mice have shown that resveratrol is able to reduce liver fat accumulation [7,8]. Some authors have also found this effect in human beings [9,10].

The mechanisms of action of resveratrol underlying its effect on liver steatosis are mainly a reduction in lipogenesis and/or an increase in fatty acid oxidation, very commonly associated with enhanced mitochondriogenesis [11]. However, little is known concerning the potential involvement of microRNAs (miRNAs) on changes induced by resveratrol in these metabolic pathways. 

MiRNAs are short double stranded RNAs (approximately 22 nucleotides) encoded in the genome that act post-transcriptionally to regulate protein expression. These non-coding RNAs can act directly on target mRNA transcripts binding to complementary target sites in 3′ untranslated regions (3′ UTR) of messenger RNAs (mRNAs), causing translational repression and/or mRNA destabilization. They can also act indirectly by regulating intermediate components, such as transcripts that encode transcription factors which, in turn, control the expression of downstream genes. A single miRNA can have multiple targets, acting simultaneously to regulate the post-transcriptional expression of various genes and physiological processes. Furthermore, each gene can be regulated by several miRNAs [12,13]. What is more, the expression of these miRNAs can be modified by changes induced either directly in the enzymes involved in their biogenesis process or in miRNA epigenetic modifications, or indirectly via lipoprotein-mediated miRNA delivery to cells, among others [14,15].

It has been reported in the literature that different types of polyphenols, such as proanthocyanidins or a mixture extracted from *Hibiscus sabdariffa*, are able to modify the expression of miRNA-122-5p (a liver specific miRNA and the most abundant one) and the paralogs miRNA-103-3p and miRNA-107-3p in liver [16,17,18,19]. 

In a previous study carried out by our group using this precise cohort of animals, resveratrol treatment did not reduce final body weight or liver weight. No changes were observed in food intake. By contrast, resveratrol treatment induced a significant decrease in hepatic triacylglycerol content. Moreover, when the activity of several enzymes involved in hepatic lipid metabolism was measured, no changes in fatty acid synthase (FAS) activity and an increase in carnitine palmitoyltransferase 1 (CPT1) activity were found, suggesting that the delipidating effect was due, at least in part, to increased fatty acid β-oxidation, which reduces the availability of fatty acids for triacylglycerol synthesis [20].

In this context, the aim of the present study was to determine whether, as in the case of other polyphenols, this reduction in liver fat was mediated by changes in the expression of miRNA-122-5p, miRNA-103-3p and miRNA-107-3p, which represent more than 75% of total miRNAs in the liver [19,21,22,23,24,25], as well as in their target genes.

## 2. Material and Methods 

### 2.1. Animals and Experimental Design

The experiment was conducted with 16 male Sprague-Dawley rats purchased from Harlan Ibérica (Barcelona, Spain) and took place in accordance with the institution’s guide for the care and use of laboratory animals (CUEID CEBA/30/2010). The rats were individually housed in polycarbonate metabolic cages (Techniplast Gazzada, Guguggiate, Italy) and placed in an air-conditioned room (22 ± 2 °C) with a 12 h light-dark cycle. After a 6-day adaptation period, rats were randomly divided into two dietary groups of eight animals each, namely a control group (Control) and a group treated with resveratrol (Resveratrol), both fed a commercial obesogenic diet (4.6 kcal/g; 44.8% energy from fat, 36.2% from carbohydrates and 19.0% from proteins) supplied by Harlan Ibérica (TD. 06415) for 6 weeks (Figure S1). Resveratrol, supplied by Monteloeder (Elche, Spain), was added to the diet as previously reported [6]. Briefly, the phenolic compound was dissolved in an ethanolic solution (5 mg/mL) and poured on the surface of the diet. Rats started eating immediately once the diet was daily replaced, and thus they ate all the resveratrol added before it started to degrade (3 h). In order to ensure a dose of 30 mg resveratrol/kg body weight/day, the amounts of resveratrol to be included in the diet for each animal were calculated daily based on their individual body weight. This dose was selected for this experiment because in a previous study we observed that, under our experimental conditions, 30 mg/kg body weight/day was the most effective of the following: 6, 15, 30 and 60 mg/kg body weight/day. Diet for control animals was added with the same amount of ethanolic solution without resveratrol. All animals had free access to food and water. Food intake and body weight were measured daily. This cohort of animals had been previously used in another study reported by our group [20,26].

At the end of the experimental period, animals were sacrificed under anaesthesia by intraperitoneally administering 400 mg chloral hydrate/kg body weight, by cardiac exsanguination, after a 12-h fasting period. The liver was dissected, weighed and immediately frozen at −80 °C. 

### 2.2. Cell Culture 

AML12 (Alpha Mouse Liver 12) hepatocytes, supplied by ATCC (ATCC CRL-2254), were cultured in 1:1 DMEM/HAM’S F12 glutamax medium containing 10% fetal bovine serum (FBS), 0.005 mg/mL insulin, 0.005 mg/mL transferrin, 5 ng/mL selenium, 40 ng/mL dexamethasone and 1% Penicillin/Streptomycin (10,000 U/mL). This medium was changed every two days. Cells were maintained at 37 °C in a humidified 5% CO_2_ atmosphere.

### 2.3. MicroRNA Expression Analysis

Total miRNAs were extracted using E.Z.N.A. miRNA kit (R7034-02; Omega Bio-Tek, Norcross, GA, USA) according to the manufacturer’s instructions. Total RNA (9 ng) was reverse-transcribed using the TaqMan MicroRNA Reverse Transcription kit (Applied Biosystems, Foster City, CA, USA), as previously reported in Gracia et al. [27]. The targeted miRNA assay sequences were as follows (source miRBase):

rno-miRNA-103-3p: 5′-AGCAGCAUUGUACAGGGCUAUGA-3′
rno-miRNA-107-3p: 5′-AGCAGCAUUGUACAGGGCUAUCA-3′
rno-miRNA-122-5p: 5′-UGGAGUGUGACAAUGGUGUUUG-3′

PCR was performed in an iCycler™–MyiQ™ Real-time PCR Detection System (Applied Biosystems, Foster City, CA, USA). Amplification was performed at 95 °C for 10 min, followed by 40 cycles of 95 °C for 15 s and 60 °C for 1 min. U6 small nuclear RNA was used as an endogenous control. All mRNA levels were normalized to the values of U6 snRNA. The results were expressed as fold changes of threshold cycle (Ct) value relative to controls using the 2^−ΔΔCt^ method [28].

### 2.4. Target Genes for miRNAs

In order to obtain the predicted and validated target genes for these miRNAs, a comparative analysis was carried out in miRecords. This database is an integrated resource for animal miRNA-target interactions, which stores predicted miRNA targets produced by 11 established miRNA target predicted programs [29]. No validated target genes were found. Among the predicted target genes, only those involved in hepatic lipid metabolism (*srebf1, fasn, cpt1a*) were selected (Table 1). *Fasn* codifies for fatty acid synthase, a key enzyme involved in de novo lipogenesis, *srebf1* is the transcription factor that regulates this enzyme, and *cpt1a* codifies for carnitine palmitoyltransferase, a key enzyme involved in the fatty acid oxidation. In addition, we reviewed the literature and found that several authors had proposed *srebf1* and *fasn* as target genes for miR-122-5p and miR-107-3p, respectively (Table 1). 

### 2.5. miRNA Transfection

Hepatocytes in a confluence status of approximately 90%, were transfected with Lipofectamine RNAiMAX (Applied Biosystems, Foster City, CA, USA) prepared following the manufacturer’s protocol, with mirVana miRNA mimics of mmu-miRNA-103-3p, mmu-miRNA-107-3p and mmu-miRNA-122-5p (homologous to rno-miRNA-103-3p, rno-miRNA-107-3p and rno-miRNA-122-5p respectively) (Applied Biosystems, Foster City, CA, USA). Each mimic was transfected for 48 h in a final concentration of 25 nM per well. Optimal transfection conditions were determined in previous experiments, and transfection efficiency was assessed using miRNA probes and fluorescent transfection controls. To rule out unspecific effects, control cells were transfected with negative controls. 

### 2.6. Western Blot Analysis 

#### 2.6.1. Liver Protein Expression of Fatty Acid Synthase, Sterol Regulatory Element-Binding Protein 1 and Carnitine Palmitoyltransferase 1a

Fatty acid synthase (FAS) and sterol regulatory element-binding protein 1 (SREBP1) protein extraction was carried out with 100 mg of liver as previously described [33]. The protein concentration was measured by bicinchoninic acid (BCA) protein assay kit (Thermo Scientific, Wilmington, DE, USA).

Immunoblot analyses were performed in all tissue samples using 80 µg of protein for FAS and 40 µg of protein for SREBP1. Protein were separated by electrophoresis in a 7.5% sodium dodecyl sulfate (SDS)-polyacrylamide gel and transferred to polyvinylidene difluoride (PVDF) membranes. Equal loading of proteins was confirmed by staining the membranes with Coomassie Blue or incubating these membranes with polyclonal mouse β-actin antibody. The membranes of the two assays were blocked with casein phosphate buffered saline (PBS)-Tween buffer for 2 h. These membranes were incubated overnight at 4 °C with mouse origin FAS immunoglobulin G (IgG) (1:1000) and SREBP1 IgG monoclonal antibodies (1:1000) (Santa Cruz Biotechnology, Santa Cruz, CA, USA). Afterwards, in both cases, new incubation with goat- anti-mouse IgG-Horseradish Peroxidase (HRP) antibody (1:5000) (Sigma, St. Louis, MO, USA) was carried out for 2 h at room temperature. Antibodies were visualized by using a chemiluminescent substrate (Thermo Scientific, Wilmington, DE, USA) and quantified by a ChemiDoc MP imaging system (BioRad, Hercules, CA, USA). After stripping*,* FAS protein-containing membranes were incubated with a polyclonal mouse β-actin antibody (1:5000) followed by goat- anti-mouse IgG-HRP antibody (1:5000) (Sigma, St. Louis, MO, USA), and measured again. The FAS protein measurements were normalized by β-actin.

For carnitine palmitoyltransferase 1a (CPT1a), 100 mg of liver were homogenized in a PBS buffer with protease inhibitors (pH 7.4) and centrifuged (14,000× *g*, 1 min, 4 °C). The pellet was resuspended in 100 µL of radioimmunoprecipitation assay buffer (RIPA buffer). The homogenates were centrifuged at 36,000× *g* for 10 min at 4 °C. The protein concentration was measured by BCA protein assay kit (Thermo Scientific, Wilmington, DE, USA).

Immunoblotting was performed after immunoprecipitation. A total of 250 μg of liver extracts were diluted with three volumes of PBS (with added protease inhibitors). CPT1a was immunoprecipitated with 1 μL of monoclonal mouse anti-CPT1a antibody (ABCAM, Cambridge, MS, USA) in constant rotation, at 4 °C, overnight. Afterwards, 20 μL Protein G Agarose (Santa Cruz Biotech, Santa Cruz, CA, USA) was added to each sample, and these were rotated for 3 h at 4 °C. The immunoprecipitated tissue samples were then washed three times with 500 μL PBS buffer. A total of 30 μg of extracts were separated by electrophoresis in a 7.5% SDS–polyacrylamide gel and then transferred to a PVDF membrane. The membranes were incubated overnight at room temperature with mouse anti-CPT1a antibody (1:1000) (ABCAM, Cambridge, MS, USA). Afterwards, polyclonal goat- anti-mouse IgG-HRP antibody (1:2500) (Sigma, St. Louis, MO, USA) was incubated for 2 h at room temperature. Antibody was visualized by using a chemiluminescent substrate (Thermo Scientific, Wilmington, DE, USA) and quantified by a ChemiDoc MP imaging system (BioRad, Hercules, CA, USA).

#### 2.6.2. SREBP1, FAS and CPT1a Protein Expression after Over-Expression in AML12

In the case of AML12 cells, total protein was extracted with 200 µL of lysis buffer as previously reported [27]. Protein concentration was measured by BCA protein assay kit (Thermo Scientific, Wilmington, DE, USA).

For FAS protein, 65 µg of cell protein extract were used to perform the immunoblotting. Protein were separated by electrophoresis in a 7.5% SDS-polyacrylamide gel and transferred to PVDF membranes. The membranes were blocked with casein PBS-Tween buffer for 2 h. These membranes were incubated overnight at 4 °C with mouse origin FAS IgG (1:1000) (Santa Cruz Biotechnology, Santa Cruz, CA, USA). Afterwards, new incubation with goat- anti-mouse IgG-HRP antibody (1:5000) (Sigma, St. Louis, MO, USA) was carried out for 2 h at room temperature. Antibodies were visualized by using a chemiluminescent substrate (Thermo Scientific, Wilmington, DE, USA) and quantified by a ChemiDoc MP imaging system (BioRad, Hercules, CA, USA). Coomassie Blue staining of membranes was used as protein loading control. In case of CPT1a and SREBP1, immunoblotting after immunoprecipitation was performed. A total of 40 μg for CPT1a and 70 μg for SREBP1 of cell extracts were immunoprecipitated. The total amount of protein was used for immunoblotting in both cases and following the same conditions as described above.

### 2.7. Statistical Analysis

Results are presented as median ± standard deviation. Statistical analysis was performed using IBM SPSS Statistics 24.0 (SPSS Inc., Chicago, IL, USA). All of the parameters are normally distributed according to the Shapiro-Wilk’s test. Student’s *t*-test was used for comparisons between both experimental groups. Significance was assessed at the *p* < 0.05 value.

## 3. Results

### 3.1. Cell Culture Studies 

**Figure 1:**
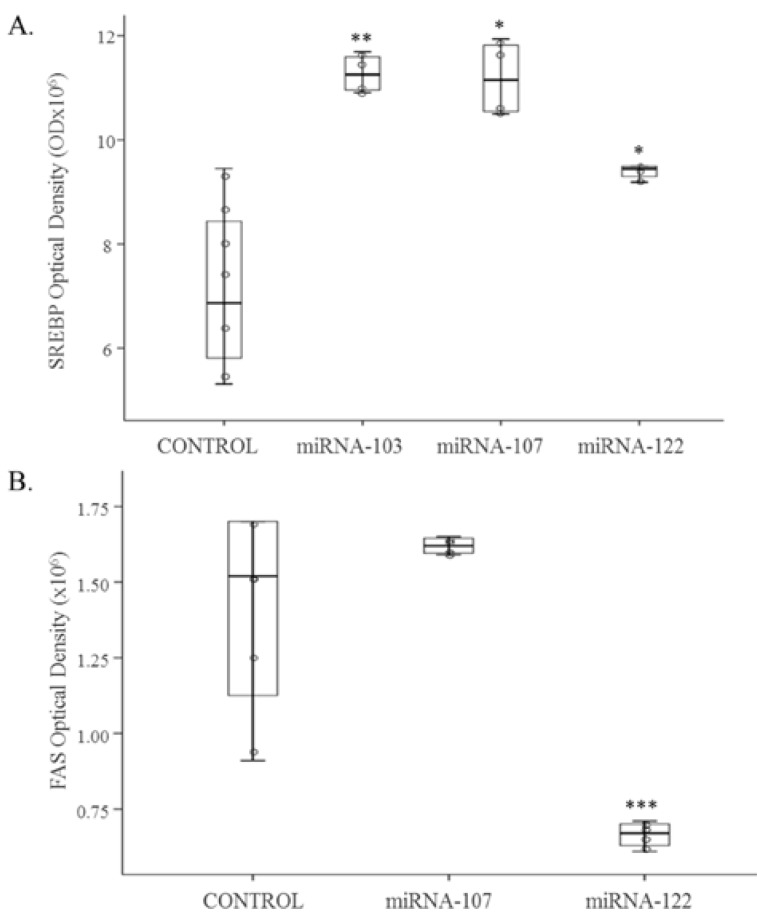
Protein expression of SREBP1 (**A**) and FAS (**B**) in AML12 control cells (*n* = 6) and AML12 cells over-expressing mmu-miRNA-103-3p, mmu-miRNA-107-3p and mmu-miR-122-5p (*n* = 6). Scatter dot plots including median and standard deviation were expressed as optical density. Comparisons between each treatment and the controls were analysed by Student’s *t*-test * *p* < 0.05, ** *p* < 0.01, *** *p* < 0.001. SREBP1: sterol regulatory element-binding protein 1, FAS: fatty acid synthase; AML12: alpha mouse liver 12.

**Figure 2:**
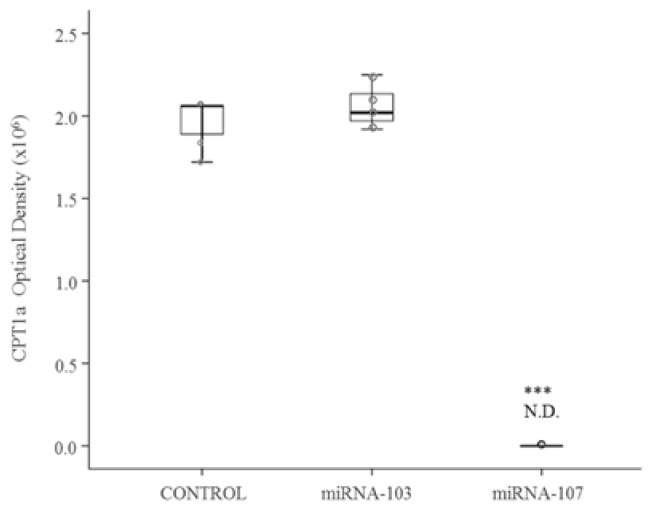
Protein expression of CPT1a in AML12 control cells (*n* = 6) and AML12 cells over-expressing mmu-miRNA-103-3p and mmu-miRNA-107-3p (*n* = 6). Scatter dot plots including median and standard deviation were expressed as optical density. Comparisons between each treatment and the controls were analysed by Student’s *t*-test. Coomassie Blue staining was used as protein loading control. ND: not detectable. CPT1a: carnitine palmitoyltransferase 1a; AML12: alpha mouse liver 12.

MiRNA-103-3p, miRNA-107-3p and miRNA-122-5p were individually over-expressed in AML12 hepatocytes. Over-expressions were confirmed by measuring each miRNA expression. Protein expression of SREBP1 was significantly increased after transfection of each miRNA (*p* < 0.05) (Figure 1A). In the case of FAS, protein expression was significantly decreased after transfection of miRNA-122-5p (*p* < 0.001) (Figure 1B). Finally, CPT1a protein expression was down-regulated by the over-expression of miRNA-107-3p (*p* < 0.001) (Figure 2). 

### 3.2. In Vivo Study

Body weight gain in rats treated with resveratrol was similar to that observed in control animals (data previously reported in Alberdi et al. 2011 [34]). Similarly, no significant differences were observed in liver weight, expressed as a percentage of final body weight (3.4 ± 0.1 in Control group and 3.5 ± 0.2 in Resveratrol group).

**Figure 3:**
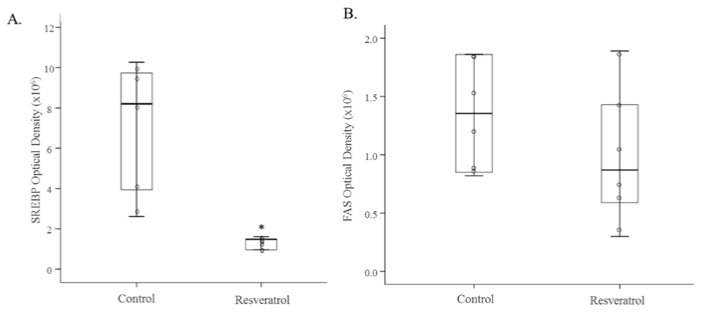
Protein expression of SREBP1 (**A**) and FAS (**B**) in the liver of rats fed an obesogenic diet supplemented with resveratrol (Resveratrol group) or not (Control group) for 6 weeks (*n* = 8). Scatter dot plots including median and standard deviation were expressed as optical density. * *p* < 0.05. Coomassie Blue staining was used as protein loading control for SREBP1 and β-actin for FAS. SREBP1: sterol regulatory element-binding protein 1, FAS: fatty acid synthase.

**Figure 4:**
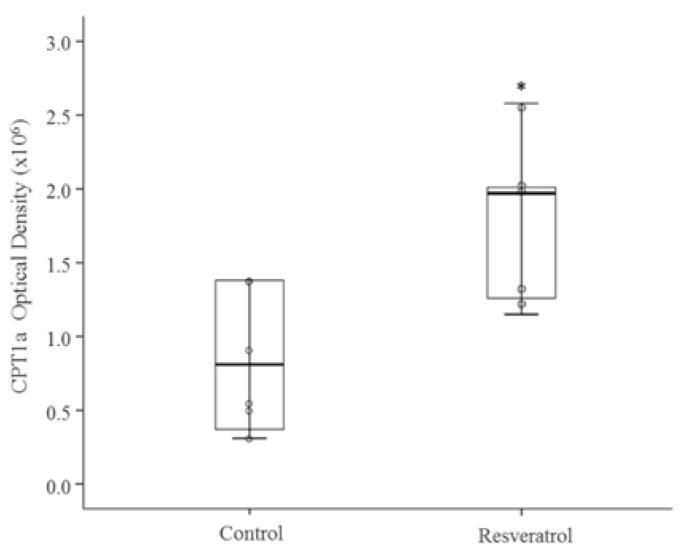
Protein expression of CPT1a in the liver of rats fed an obesogenic diet supplemented with resveratrol (Resveratrol group) or not (Control group) for 6 weeks (*n* = 8). Scatter dot plots including median and standard deviation were expressed as optical density. * *p* < 0.05. Coomassie Blue staining was used as protein loading control. CPT1a: carnitine palmitoyltransferase 1a

In the present study, we observed that the expression of the three miRNA analysed (miRNA-103-3p, miRNA-107-3p and miRNA-122-5p) was significantly reduced in the liver of rats treated with resveratrol (Table 2). When protein expression of the target genes for these miRNAs was measured, we observed a significant reduction in SREBP1 (*p* < 0.05) and a significant increase in CPT1a (*p* < 0.05) in resveratrol-treated rats (Figure 3A and Figure 4). No changes were found in FAS protein levels (Figure 3B). 

## 4. Discussion

As indicated in the Introduction, in a previous study we had observed that resveratrol was able to partially prevent liver steatosis induced by an obesogenic diet. We found a significant increase in the activity of CPT1a, a rate-limiting enzyme in fatty acid oxidation, in the liver of rats treated with resveratrol, without changes in FAS activity [20]. The present study helps us to gain more insight into the effect of resveratrol on the regulation of these two enzymes.

As far as the lipogenic pathway is concerned, miRecords data base showed that *srebf1* was a predicted target gene for miRNA-103-3p and miRNA-107-3p and *fasn* for miRNA-122-5p. In addition, Iliopoulos et al. [32] showed that the up-regulation of miRNA-122 induced the increased protein expression of SREBP1. Taking into account that miRNA are negative regulators of protein translation and that no miRNA-122-5p binding sites are found in the 3′UTR or the coding region of this gene, the authors suggested that miRNA-122 could regulate other genes that, in turn, could affect the transcription of *srebf1*. They concluded that *srebf1* was an indirect target gene for miRNA-122, but they did not describe the intermediate steps in the signaling cascade that led to the up-regulation of SREBP1. Later on, Shibata et al. [31] reported that silencing miRNA-122 led to decreased SOCS3 expression, which in turn increased STAT3 expression. Therefore, SREBP1 was negatively regulated by STAT3 and, consequently, a decrease in miRNA-122 induced a reduction in SREBP1 expression. In order to obtain more scientific support concerning the involvement of these three miRNAs in SREBP1 regulation, in the present study we over-expressed these miRNAs in AML12 hepatocytes. In all the three cases we observed a significant increase in SREBP1 protein expression. 

In rats treated with resveratrol, we found a significantly decreased expression of miRNA-103-3p, miRNA-107-3p and miRNA-122-5p, which was paralleled by a significant decrease in SREBP1 protein expression. As far as miRNA-122-5p is concerned, taking into account the results of our transfection study, and bearing in mind the results reported by Iliopoulos et al. and Shibata et al. [32,31], it can be proposed that resveratrol decreases the protein expression of the transcription factor SREBP1 indirectly via miRNA-122-5p.

With regard to miRNA-103-3p and miRNA-107-3p, as indicated before in the Discussion section, computational analysis (miRecords) revealed complementarity between these miRNAs and the 3′UTR region of *srebf1*, suggesting that it can be a direct target gene. Usually, miRNAs regulate gene transcription in a negative way, which is to say that they inhibit this process. However, in some cases, the transcription of the RNAs is positively regulated and thus, the up-regulation of some miRNAs increases mRNA levels of their targets [35]. In our in vitro study, the over-expression of miRNA-103-3p and miRNA-107-3p in hepatocytes led to an increased expression of SREBP1, suggesting that in fact they were positive regulators. In the in vivo study, resveratrol induced the down-regulation of miRNA-103-3p and miRNA-107-3p in the liver, which was accompanied by a reduced expression of SREBP1. Taking all that into account, it may be said that miRNA-103-3p and miRNA-107-3p are involved as positive regulators in the effects of this polyphenol on SREBP protein expression [35].

As shown in Table 1, *fasn* was a predicted target gene only for miRNA-122-5p. Bhatia et al. [30] transfected HepG2 hepatocytes with miRNA-107 at various doses and they observed that, using the most common dose in transfection studies (25 nM), no changes in FAS protein expression were observed. When we measured FAS protein expression, we found no change in resveratrol-treated rats. This result is in good accordance with the lack of change in FAS activity observed in our previous study addressed to this cohort of rats. However, it was somewhat surprising because *fasn* is, according to the miRecords data base, a predicted target gene for miRNA-122-5p, which was reduced by resveratrol, and in fact our transfection experiment showed that the over-expression of miRNA-122-5p induced a significant reduction in FAS protein expression. Consequently, increased FAS expression should be expected. On the other hand, SREBP1, which is a transcription factor that regulates FAS, was reduced in resveratrol-treated rats. Thus, it could be hypothesized that the increase in FAS protein expression expected as a consequence of miRNA-122-5p down-regulation could be compensated by the decrease expected due to the reduction in SREBP1.

According to the miRecords data base, *cpt1a* is a predicted target gene for miRNA-103-3p and miRNA-107-3p. In cultured hepatocytes, we observed that only those over-expressing miRNA-107-3p showed the down-regulation of CPT1a protein expression, suggesting that in fact *cpt1a* is a real target gene for miRNA-107-3p, but not for miRNA-103-3p. In our in vivo experiment, rats treated with resveratrol showed decreased miRNA-107-3p expression and increased CPT1a protein expression. All in all, these results suggest that the increase induced by resveratrol in CPT1a protein expression, which is involved in the liver delipidating effects of this polyphenol, was mediated by a reduction in miRNA-107-3p expression.

## 5. Conclusions

The present study provides new evidence showing that *srebf1* is a target gene for miRNA-103-3p and miRNA-107-3p and *cpt1a* a target gene for miRNA-107-3p. Furthermore, the reduction in liver steatosis induced by resveratrol under our experimental conditions is mediated, at least in part, by increased CPT1a protein expression and activity, via a decrease in miRNA-107-3p expression.

## Figures and Tables

**Table 1:** Predicted target genes and validated genes reported in the literature related to triacylglycerol metabolism of the miRNAs studied.

miRNA	Predicted Target Genes (miRecords)	Data from the Literature
rno-miR-103-3p	*Srebf1* *Cpt1a*	
rno-miR-107-3p	*Srebf1* *Cpt1a*	*Fasn*: Bhatia et al. [30]
rno-miR-122-5p	*Fasn*	*Srebf1*: Shibata et al. [31] *Srebf1*: Iliopoulos et al. [32]

*Srebf1*: sterol regulatory element binding factor 1; *Cpt1a*: carnitine palmitoyltransferase 1a; *Fasn*: fatty acid synthase.

**Table 2:** The gene expression fold change of miRNA-103, miRNA-107 and miRNA-122 in the liver of rats fed an obesogenic diet supplemented with resveratrol (Resveratrol group) or not (Control group) for 6 weeks (*n* = 8).

miRNA	Fold Change (Resveratrol vs. Control)	*p*
miR-103	−2.49	<0.01
miR-107	−2.08	<0.05
miR-122	−2.59	<0.01

## Supplementary Material

The following are available online at www.mdpi.com/2072-6643/9/4/360/s1, Figure S1: Diagram of the work-plan for the in vivo study.

## Abbreviations

3′UTR	3′ untraslated regions
BCA	bicinchoninic acid
CPT1a	carnitine palmitoyltransferase 1a
FAS	fatty acid synthase
FBS	fetal bovine serum
miRNA	microRNA
mRNA	messenger RNA
SREBF1	sterol regulatory element binding factor 1
SREBP1	sterol regulatory element binding protein 1
