# Supplementary material for: Are miRNA-103, miRNA-107 and miRNA-122 Involved in the Prevention of Liver Steatosis Induced by Resveratrol?
Source: Nutrients. 2017 Apr 4;9(4):360. doi: 10.3390/nu9040360 (PMC5409699; doi:10.3390/nu9040360)
Supplement: Supplementary file 1 [file nutrients-09-00360-s001.pdf]

Supplementary Material: Are miRNA-103, miRNA-107 and miRNA-122 Involved in the Prevention of Liver Steatosis Induced by Resveratrol?

Ana Gracia, Alfredo Fernández-Quintela, Jonatan Miranda, Itziar Eseberri,
Marcela González and María P. Portillo

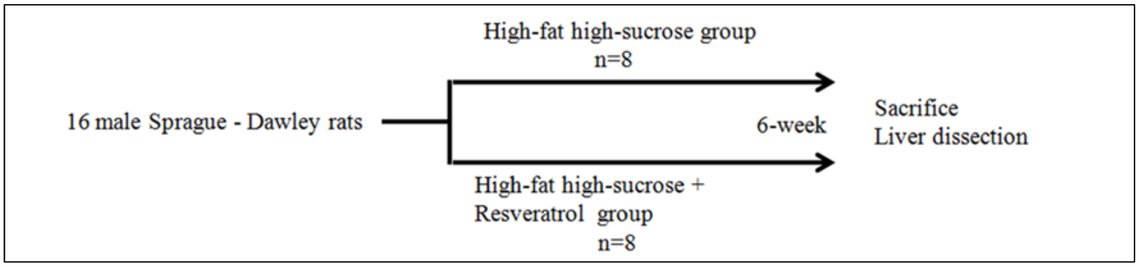

Figure S1. Diagram of the work-plan for the in vivo study.

© 2017 by the authors. Licensee MDPI, Basel, Switzerland. This article is an open access article distributed under the terms and conditions of the Creative Commons by Attribution (CC-BY) license (http://creativecommons.org/licenses/by/4.0/).
